# Microtia: A Data Linkage Study of Epidemiology and Implications for Service Delivery

**DOI:** 10.3389/fped.2021.630036

**Published:** 2021-03-26

**Authors:** Thomas H. Jovic, John A. G. Gibson, Rowena Griffiths, Thomas D. Dobbs, Ashley Akbari, Nicholas Wilson-Jones, Rhodri Costello, Peter Evans, Mark Cooper, Steve Key, Ronan Lyons, Iain S. Whitaker

**Affiliations:** ^1^The Welsh Centre for Burns and Plastic Surgery, Morriston Hospital, Swansea, United Kingdom; ^2^Reconstructive Surgery and Regenerative Medicine Research Group (ReconRegen), Swansea University Medical School, Institute of Life Sciences, Swansea, United Kingdom; ^3^Health Data Research UK, Swansea University, Swansea, United Kingdom; ^4^Administrative Data Research Wales, Swansea University, Swansea, United Kingdom; ^5^Department of Otolaryngology, Morriston Hospital, Swansea, United Kingdom; ^6^Department of Maxillofacial Surgery, Morriston Hospital, Swansea, United Kingdom

**Keywords:** microtia, epidemiology, reconstructive surgery, congenital, otology

## Abstract

**Introduction:** Previous studies of microtia epidemiology globally have demonstrated significant geographical and ethnic variation, cited broadly as affecting 3–5 in 10,000 live births. The aim of this study was to determine the incidence of microtia in a largely homogeneous ethnic population in the United Kingdom (Wales) and to identify factors, such as distance and socioeconomic status, which may influence the access to surgical intervention.

**Materials and Methods:** A retrospective cohort study was conducted using data linkage to identify patients born between 2000 and 2018 with a diagnosis of microtia. Microtia incidence was calculated using annual and geographic birth rates. Surgical operation codes were used to classify patients into those that had no surgery, autologous reconstruction or prosthetic reconstruction. Sociodemographic attributes were compared using descriptive statistics to determine differences in access to each type of surgical intervention.

**Results:** A total of 101 patients were identified, 64.4% were male and the median age was 12 (8–16). The mean annual incidence was 2.13 microtia cases per 10,000 births over the 19-year study period. Both temporal and geographic variation was noted. The majority of patients undergoing surgery opted for autologous reconstruction (72.9%) at a median age of 9 (7–10) compared to 7 (5–8) for prosthetic reconstruction. Autologous reconstruction had a higher median number of surgeries (2, 1–3) than prosthetic (1.5, 1–2) and a higher median socioeconomic status of 3 (2–4) compared to 2 (1–4) for the prosthetic cohort. There were no statistically significant differences in the distance traveled for surgery.

**Discussion:** This study highlights a role for data linkage in epidemiological analyses and provides a revised incidence of microtia in Wales. Although the majority of patients opted for autologous reconstruction, demographic disparities in socioeconomic status warrant further investigation, emphasizing the importance of striving for equity in accessibility to surgical intervention.

## Introduction

Abnormalities of the ear have been evident as early as the prehistoric era (1), with the earliest documented records of surgery from the Edwin Smith Surgical Papyrus and Sushruta Samhita describing methods of reconstructing the traumatized auricle (2, 3). Contemporary ear reconstruction practices have evolved to encompass the spectrum of congenital defects known as microtia, the presence of a smaller and usually malformed auricle, or anotia in which the auricle is absent in its entirety. Schiemeden first described auricular reconstruction using costochondral grafts in 1908 (4), with incremental refinements over the past century by individuals such as Tanzer, Brent, Nagata, and Firmin (5–8). The latter two approaches now dominate autologous reconstructive practice worldwide (9). Alternatives to autologous reconstruction include the use of prosthetics, which have emerged from the advent of osseointegrated prosthesis retention by Branemark in 1977 (10) and synthetic materials such as Medpor described by Reinisch in 1991 (11).

Microtia can present as an isolated phenotype or as a manifestation of genetic syndromes such as Hemifacial Microsomia, Treacher Collins or Goldenhar Syndrome. In addition to its aesthetic impact, it can also affection function via meatal atresia and hearing loss (12, 13). Microtia can therefore be associated with significant functional and psychosocial issues, such as impaired speech and language development (14), attention deficit disorders (15), and psychological implications for both the patient (16) and their careers (17).

An appreciation of the epidemiology of microtia is important, owing to the high surgical burden and follow-up costs. In an ideal healthcare system, service provision would mirror the geographical distribution and clinical need of affected patients.

Previous studies of microtia prevalence globally have demonstrated significant geographical and ethnic variation (18). Although cited broadly as affecting ~3–5 in 10,000 live births (19), higher frequencies have been noted in certain ethnic groups such as the Navajo population (12 per 10,000 births) (20) and 8.8 per 10,000 births in Chile (21). Studies have indicated a male predisposition of up to 40% higher risk, and a primarily unilateral phenotype in which the right ear is affected more frequently than the left (12, 22). In Wales the prevalence of microtia was previously reported as 1.11 per 10,000 births (18), which is slightly higher than the average rate for Western Europe (0.88 per 10,000 births).

In the United Kingdom (UK), surgical options for microtia patients include both alloplastic and autologous reconstruction, with the choice determined through discussions between the patient, parents and clinicians from birth until 8 to 10 years of age (9, 23). At this age, the auricle is ~90% of its adult size and children will generally have acquired sufficient self-awareness and psychosocial experience to contribute to the decision-making process (24, 25). The rib cage is also developed to a suitable size to support carving of the auricular framework. Both autologous and alloplastic reconstruction have been previously demonstrated to enhance quality of life of patients with microtia and anotia (26) and in expert hands, offer excellent results with relatively low complication rates (23). In the UK, plastic surgery is a tertiary service, with auricular reconstruction offered at a handful of specialist centers. Accessibility to, and awareness of, these procedures may well be influenced by geographic and social factors.

The aim of this study was to use data linkage to identify a cohort of microtia patients in Wales and determine geographical and socioeconomic variations in microtia epidemiology and any association with the choice of autologous or prosthetic reconstruction.

## Materials and Methods

The retrospective cohort study was designed and reported in accordance with the Reporting of studies Conducted using Observational Routinely-collected health Data (RECORD) statement (27) and the STROBE statement (28).

### Overview

Analysis of routine population-scale data from primary and secondary care National Health Service (NHS) and national administrative data sources for 2000–2018 in Wales, UK (population 3.1 million) were performed. In instances where relevant data were unavailable from a single source, multiple data sources were linked. Data was retrieved from six national data sources (Table 1). In Wales, population level de-identified person-based health and socio-economic administrative data are collated and linked within the Secure Anonymized Information Linkage (SAIL) Databank (29–31). Robust policies, structures, and controls are in place to protect privacy through a reliable matching and anonymization process, achieved in conjunction with the NHS Wales Informatics Service (NWIS) using a split file multiple encryption approach described in detail in previous published work (30).

**Table 1:** List of databases used for data linkage and their descriptions.

**Database**	**Description**
Annual District Death Extract (ADDE)	Collected from the Office for National Statistics (ONS), this database contains death registration information, relating to Welsh Residents including those who died outside of Wales.
Outpatient Dataset for Wales (OPDW)	Administrative and clinical data obtained from outpatient appointments in Wales.
Patient Episode Database for Wales (PEDW)	Administrative and clinical data for all hospital admissions, including diagnosis and operations performed.
Welsh Cancer Intelligence and Surveillance Unit (WCISU)	The national cancer registry for Wales. Captures all welsh melanoma patients from a number of sources; Multi-Disciplinary Team data, pathology data, other routine data sources in Wales and the English cancer registry.
Welsh Longitudinal General Practice dataset (WLGP)	Administrative and clinical data from all patient visits to a General Practitioner.
Welsh Demographic Service (WDS)	Administrative data about individuals resident or registered in Wales that have used National Health Service (NHS) services.

### Study Population

Microtia patients were identified from two separate sources, the Welsh Longitudinal General Practice (WLGP) data using READ codes for microtia (P422. Microtia; P401z Absence of external ear NOS) and secondary care obtained from Patient Episode Database for Wales (PEDW) and Outpatient Dataset for Wales (OPDW) using the International Classification of Disease 10 (ICD-10) code Q17.2 Microtia.

Demographic information and socioeconomic status were retrieved. The Welsh Index of Multiple Deprivation (WIMD) version 2011, is used as the official area level measure of socioeconomic status by the Welsh Government (48). Individual scores are based upon a person's postal address. Wales is divided into 1,896 Lower layer Super Output Areas (LSOAs) following the 2001 Census, each consisting of ~1,600 people. The WIMD scores for each LSOA are calculated and weighted using data from eight domains of socioeconomic status (income, employment, health, education, access to services, community safety, physical environment, and housing socioeconomic status). Each LSOA in Wales has been ranked according to its WIMD score and grouped into quintiles, with quintile 5 being the highest socioeconomic status and 1 being the lowest socioeconomic status.

### Study Outcomes

#### Incidence

Incidence rates were calculated for each year of the study and per geographical local authority, using the number of live births recorded for Wales from the Office of National Statistics as the denominator and presented as a rate per 10,000 live births.

#### Operative Procedures

Patient Episode Database for Wales (PEDW) was used to identify all day case and hospital admissions with inpatient surgical procedures recorded. Patients were classified as receiving no auricular reconstructive surgery, prosthetic reconstructive surgery or autologous reconstructive surgery based on appropriate Office of Population Censuses and Surveys Classification of Interventions and Procedures (OPCS-4) codes.

#### Access to Healthcare

For each individual surgical procedure, Geographic Information System (GIS) software was used to calculate the geodesic distances (i.e., straight-line distance) between each patient's LSOA to the respective treatment center. To ensure data anonymity, patient addresses are not stored in SAIL. Centroid co-ordinates of each patient's LSOA were used as a proxy for their location.

### Ethical Approval

Approval for the use of anonymized data in this study, provisioned within the Secure Anonymized Information Linkage (SAIL) Databank was granted by an independent Information Governance Review Panel (IGRP) under project 0651. The IGRP has a membership comprised of senior representatives from the British Medical Association (BMA), the National Research Ethics Service (NRES), and Public Health Wales and NHS Wales Informatics Service (NWIS). Usage of additional data was granted by data owner. The SAIL Databank is General Data Protection Regulations (GDPR) and the UK Data Protection Act compliant.

The data sources used in this study are available in the SAIL Databank at Swansea University, Swansea, UK, but as restrictions apply they are not publicly available. All proposals to use SAIL data are subject to review by an independent Information Governance Review Panel (IGRP). Before any data can be accessed, approval must be given by the IGRP. The IGRP gives careful consideration to each project to ensure proper and appropriate use of SAIL data. When access has been granted, it is gained through a privacy protecting safe haven and remote access system referred to as the SAIL Gateway. SAIL has established an application process to be followed by anyone who would like to access data via SAIL at https://www.saildatabank.com/application-process.

### Statistical Analysis

Descriptive statistics (median and interquartile range) were used to characterize the microtia cases and a chi squared test was used to determine disparities between the cohorts that underwent surgical intervention. All data were analyzed using IBM SPSS Statistics for Windows (IBM Corp. Released 2017. Version 25.0. Armonk, NY: IBM Corp). Statistical significance was assumed with a *p* < 0.05.

## Results

### Demographics

Between 2000 and 2018, 101 patients in Wales were born with a diagnosis of microtia. The demographic data of this cohort is outlined in Table 2. 65 (64.4%) were recorded as being of male sex at birth.

**Table 2:** Patient Demographics assessed at the time of analysis (April 2020).

**Parameter**	**Total (*n* = 101)**	**No Operation (*n* = 53)**	**Prosthetic (*n* = 13)**	**Autologous (*n* = 35)**	***P-*value**
**Age at time of analysis (years)**, ***n*** **(%)**					
<5	9 (9)	9 (17)	0 (0.0)	0 (0)	0.00
5–10	31 (31)	26 (49)	<5 (25)	<5 (<6)	
11–15	29 (29)	8 (15)	8 (62)	13 (37)	
16–20	28 (28)	8 (15)	<5 (25)	18 (51)	
>20	<5 (<5)	<5 (<5)	0 (0)	<5 (<6)	
Median age (IQR)	12 (8–16)	8 (6–12)	11.5 (10–13)	15 (13–16.5)	
**Sex**, ***n*** **(%)**					
Male	65 (64)	36 (68)	8 (62)	21 (60)	0.73
Female	36 (36)	17 (32)	5 (39)	14 (40)	
**WIMD Quintile**, ***n*** **(%)**					
1 (lowest socioeconomic status)	21 (21)	9 (17)	5 (42)	7 (20)	0.61
2	23 (23)	13 (25)	<5 (<20)	8 (23)	
3	19 (19)	12 (23)	<5 (<20)	5 (14)	
4	20 (20)	9 (17)	<5 (<20)	10 (29)	
5 (highest socioeconomic status)	16 (16)	9 (17)	<5 (<20)	5 (14)	
Unspecified	<5 (<5)	<5 (2)	<5 (<20)	0 (0)	
Median (IQR)	3 (2–4)	3 (2–4)	2 (1–4)	3 (2–4)	
**Number of Procedures**, ***n*** **(%)**					
0	51 (51)	51 (96)	–	–	0.00^*^
1	24 (24)	<5 (<5.0)	7 (54)	15 (43)	
2	14 (14)	–	<5 (<35)	10 (29)	
3	8 (8)	–	<5 (<35)	7 (20)	
4	<5 (<5)	–	0 (0)	<5 (9)	
5	0 (0)	–	0 (0)	0 (0)	
6	<5 (<5)	–	<5 (<35)	0 (0)	
**Median number of procedures Median (IQR)**	1 (0–2)	0 (0)	1.5 (1–2)	2 (1–3)	
		^*^p = 0.29 between prosthetic and autologous cohorts			
**Median age of surgical Intervention (years)**					
Median (IQR)	8 (5–10)	–	7 (5–8)	9 (7–10)	0.02
**Distance Traveled for Surgery (miles)**, ***n*** **(%)**					
<20	23 (48)	–	5 (39)	18 (51)	0.37
20–50	12 (25)	–	<5 (<35)	8 (23)	
50–100	7 (15)	–	<5 (<35)	6 (17)	
>100	5 (10)	–	<5 (<35)	<5 (<10)	
Unspecified	<5 (<5)	–	<5 (<35)	0 (0)	
Median (IQR)	20.8 (6.9–51.5)	–	32.2 (7.9–50.4)	19.9 (6.9–51.5)	

There were no statistically significant differences in the proportion of patients within each WIMD quintile (*p* = 0.85) with a median deprivation (WIMD) score of 3 [Inter Quartile Range (IQR) 2–4]. Of the patients in this cohort, <5 (4%) had microtia as part of a congenital syndrome.

### Microtia Incidence and Geographic Distribution

**Figure 1:**
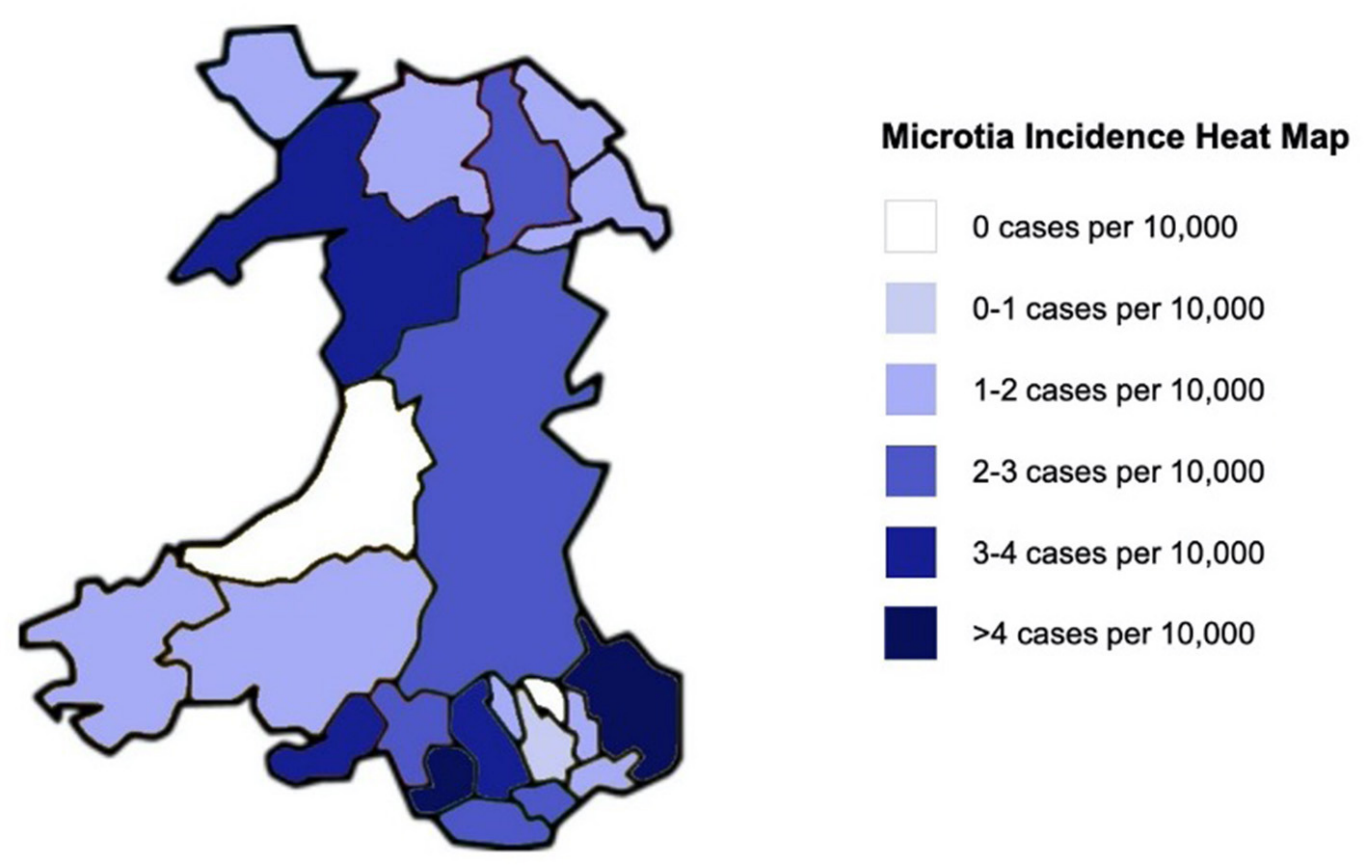
Heat map to demonstrate the regional variability in microtia incidence per 10,000 live births in Wales between 2000 and 2015.

The incidence of microtia in Wales between 2000 and 2018 was 2.13 cases per 10,000 births. Annual birth rate data per local authority area was only available from the Welsh Government between the years of 2000 and 2015 and was used to determine the geographical incidence of microtia in Wales during this time period. There was noted to be variability in the incidence of microtia throughout the different local authorities of Wales (Figure 1), with the highest incidences per 10,000 live births noted in Monmouthshire (5.54) followed by Bridgend (5.18).

Conversely, there were no reported cases of microtia in Ceredigion or Blaenau Gwent during this study period, and the lowest incidences per 10,000 live births were noted in Caerphilly (0.86) and Flintshire (1.07).

**Figure 2:**
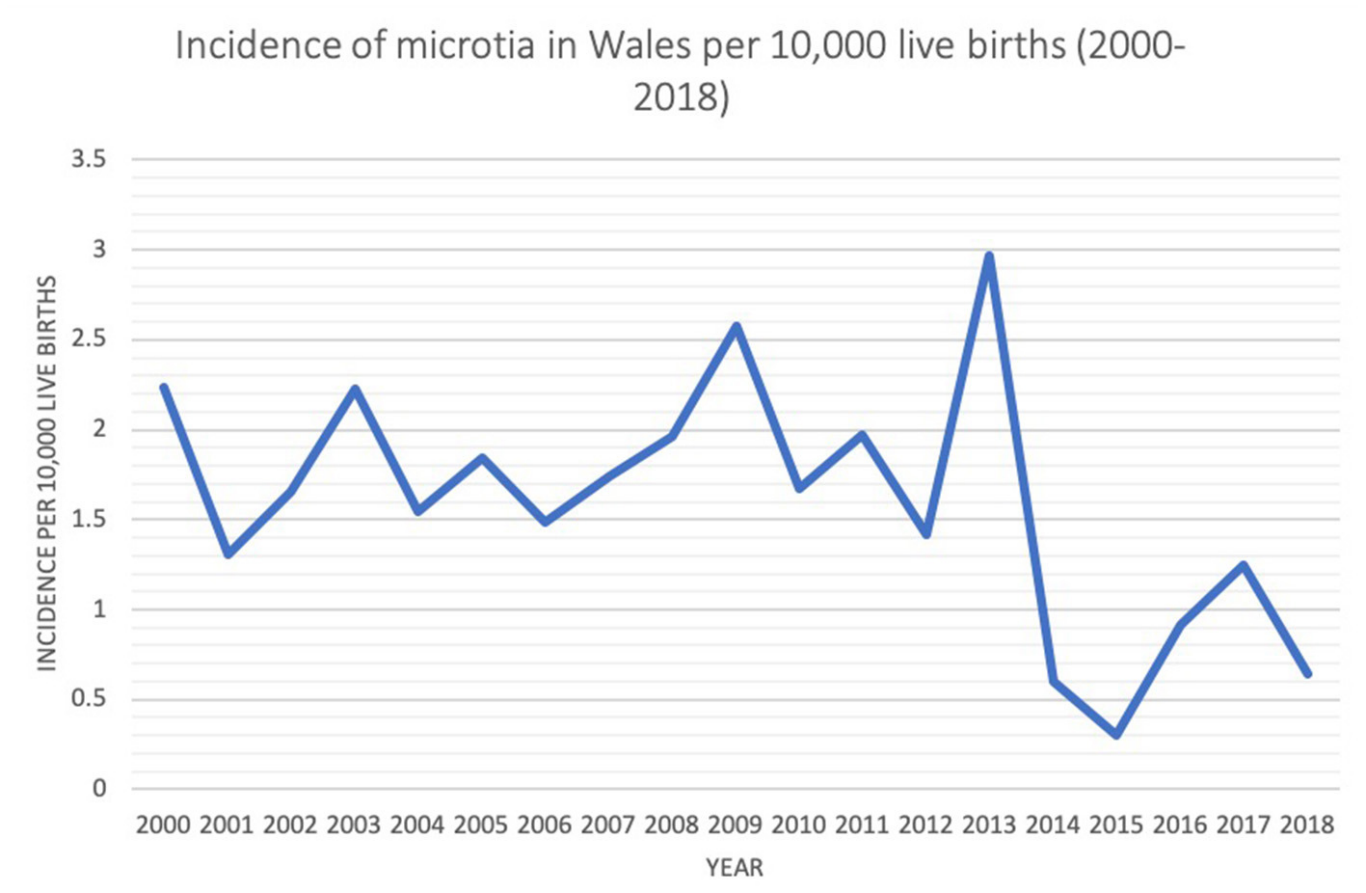
Annual incidence of microtia in Wales during study period 2000–2018.

Annual birth data for Wales as a nation was available from 2000 to 2018. During this period, there was an annual fluctuation in the incidence of microtia cases per 10,000 live births. A peak annual incidence of 2.96 was noted in 2013, with the lowest incidence of 0.3 in 2015 (Figure 2).

### Surgical Intervention: Autologous and Prosthetic Reconstruction

Of the 101 microtia patients in this cohort, 48 patients (47.5%) had auricular reconstructive surgery during the study period (2000–2018). Of the 48 patients who had reconstructive surgery, 13 (27.1%) had a record of receiving a prosthetic auricular replacement and 35 patients (72.9%) were noted to have undergone an autologous auricular reconstruction. There were no significant differences in sex or deprivation scores between the patients in the non-operative, prosthetic and autologous groups. With regard to patients undergoing reconstructive surgery, the median age of surgical intervention was higher in the autologous reconstruction cohort at 9-years of age, compared to 7-years of age in the prosthetic cohort (*p* = 0.02). WIMD score was higher (indicating less deprived) in the autologous cohort (3, IQR = 2–4) compared to the prosthetic group (more deprived; median WIMD score 2, IQR = 1–4) but this was not a statistically significant difference (*p* = 0.488).

Of the 101 patients, 53 patients had no recorded surgical intervention recorded during the study period (2000–2018). Sixty-six percent of these patients were in the younger age categories (<10 years of age), and a younger median age was seen in this cohort (8, IQR = 6–12) compared to the patients in the autologous (15, IQR = 13–16.5) and prosthetic (11.5, IQR = 10–13) reconstruction cohorts (*p* < 0.00). Fewer than 5% in the “No operation” cohort underwent procedures to the auricle, of which neither were reconstructive in nature (Table 2).

The median number of surgeries in the prosthetic group was 1.5 (IQR = 1–2) and in the autologous group was 2 (IQR = 1–3). No statistical difference was observed between the number of procedures between the prosthetic and autologous groups (*p* = 0.29).

### Geodesic Distance Traveled for Surgery

**Figure 3:**
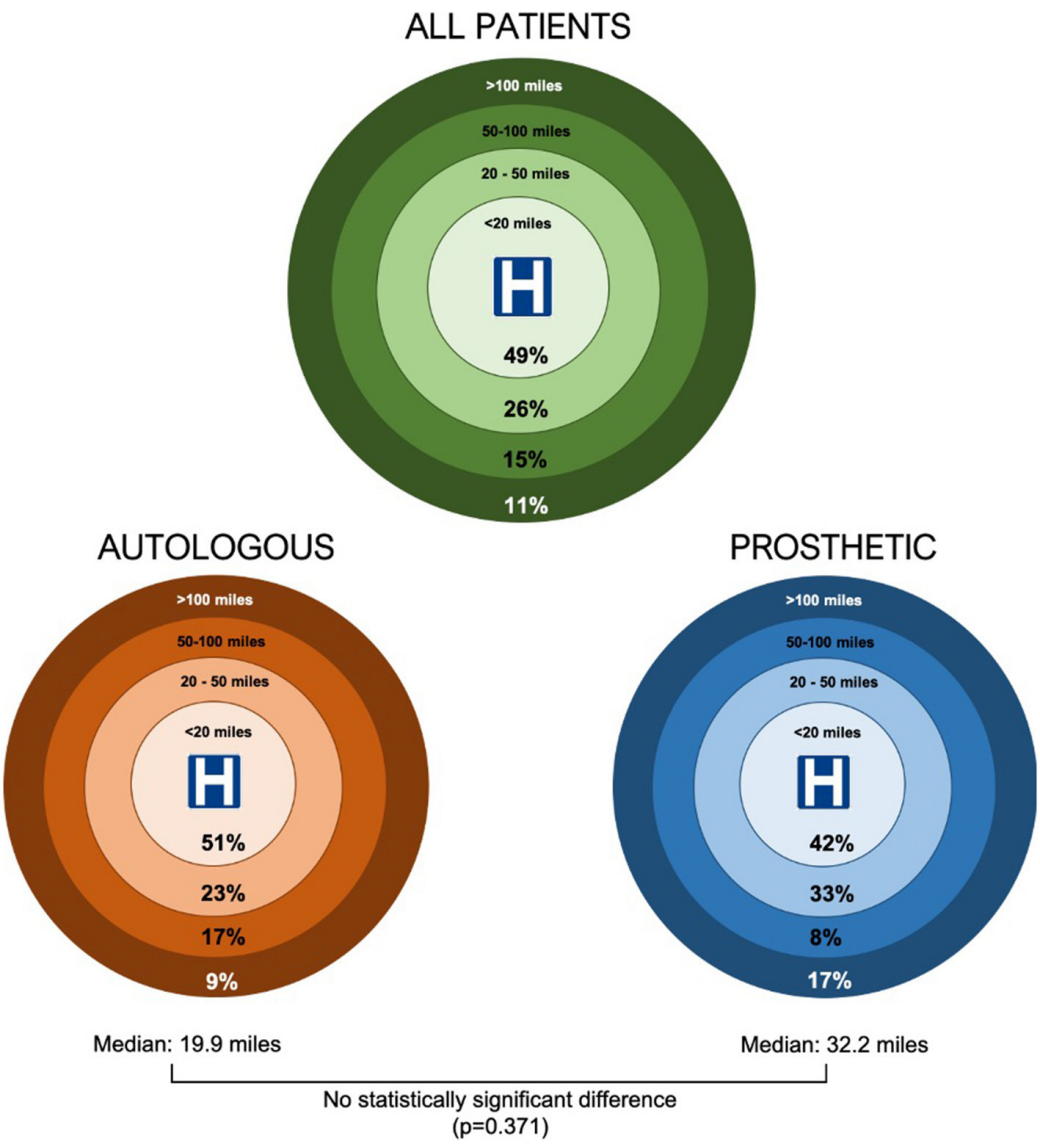
Geodesic distances traveled by patients undergoing microtia reconstructive surgery in Wales.

The median geodesic distance traveled for all patients undergoing surgical intervention was 20.81 miles (IQR = 6.9–51.5). In the autologous reconstruction cohort, a median distance of 19.86 miles was traveled for surgical intervention (IQR = 6.9–51.5 miles) and in the prosthetic group a larger median distance of 32.22 miles was traveled (IQR = 7.9–50.4). The majority (47.9%) of patients were within a 20 miles radius from their site of surgery (Figure 3). The differences in distance traveled by each cohort were not statistically significant (*p* = 0.37).

Out of the 48 patients who had surgical intervention, 10 (20.8%) had procedures undertaken in England with a median geodesic distance of 104.2 miles to the site of surgery, of which 6 (60%) of these cases were autologous reconstructions. There were no cases known to be performed outside of the United Kingdom. Of the 38 cases (79.2%) performed in Wales, 31 cases were done in The Welsh Center for Burns and Plastic Surgery, Morriston Hospital, Swansea, and 7 in other Welsh hospitals.

**Figure 4:**
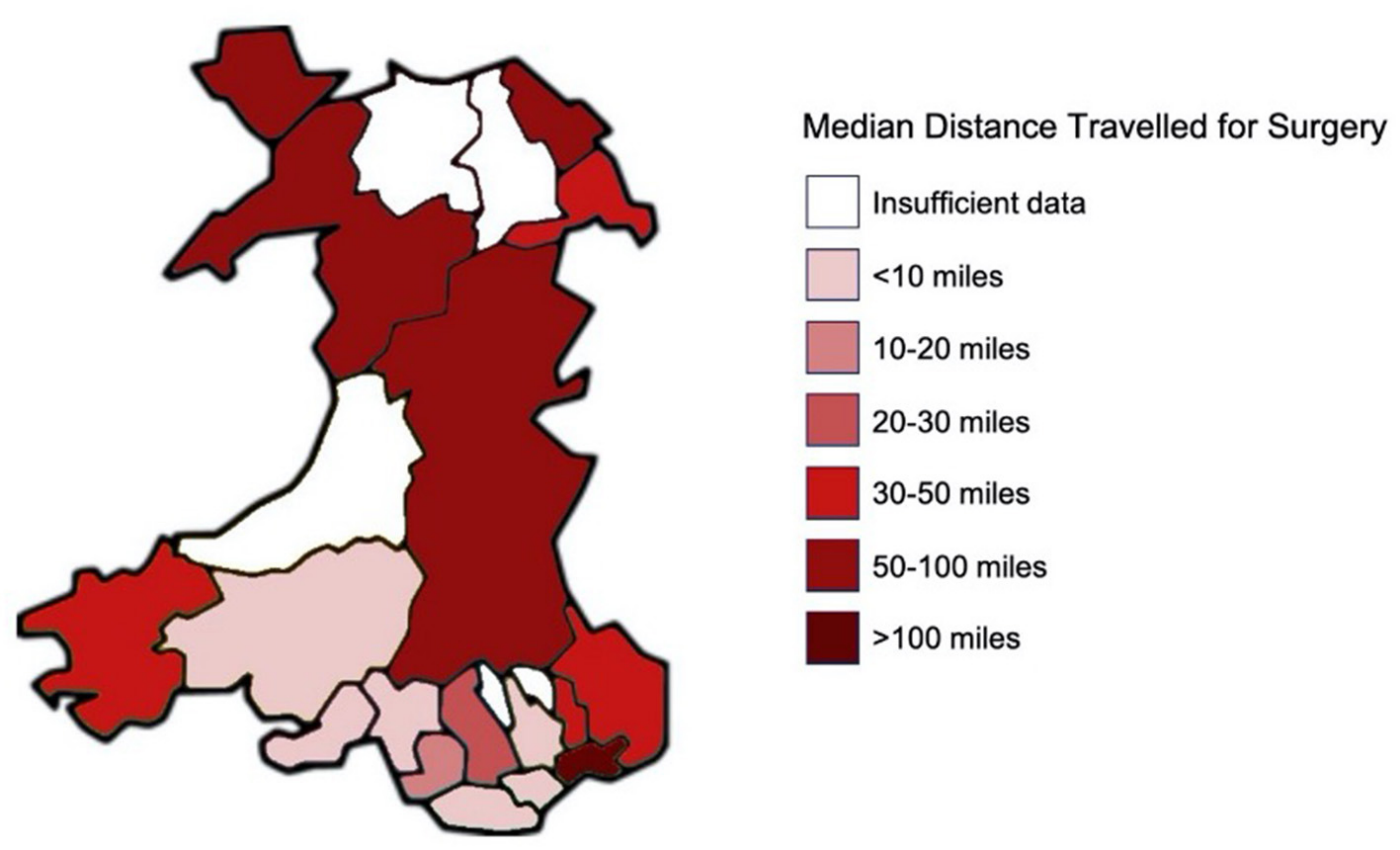
Heat map to demonstrate the regional variability in geodesic distance traveled for surgery in Wales.

There was noted to be geographical variation in the median distance traveled for surgery throughout Wales (Figure 4). Patients located in the peripheries of the country such as Powys (83.0 miles), Newport (125.2 miles), Anglesey (83.3 miles), and Flintshire (91.4 miles) were amongst the highest median geodesic distances traveled. There was no statistically significant association between WIMD quintile and distance traveled (*p* = 0.07).

## Discussion

This study is the first to our knowledge to detail the regional epidemiology of microtia in a large homogenous population in the United Kingdom (3.1 million) including the association between incidence, socioeconomic deprivation, and access to reconstructive surgery.

Previously published epidemiological data derived from the International Clearinghouse for Birth Defects Surveillance and Research database calculated the incidence of microtia in Wales to be 1.11 per 10,000 births between 1998 and 2007 (18). Our study provides a revised incidence statistic of 2.13 per 10,000 births based on a broader, more recent period of 19 years (2000–2018). The more accurate detection rate of more than double the number microtia diagnoses in this study is via the use of data linkage to enable the retrieval of information from a combination of secondary care inpatient and outpatient data, in addition to primary care GP records. Although the overall incidence is comparable to epidemiological studies of predominantly Caucasian populations in California, US (2.2 per 10,000 births) and Sweden (2.4 per 10,000 births), it is significantly higher than the published rates for England (0–0.7 per 10,000 births) and quoted Western European rates as a whole (0.88 per 10,000 births) (18, 32, 33). The sex ratio in our cohort (64% Male: 36% Female) is in line with contemporary European data from Finnish (34) and German studies (35), in addition to heterogenous ethnic populations in Japan (15), USA (36), Venezuela (37), and Mexico (38).

The incidence of microtia in Wales displayed geographic variability, with a difference in incidence >4-fold observed between local authorities. The highest incidences of microtia was in South-Eastern areas of Wales such as Bridgend and Monmouthshire (>4 per 10,000 births), with relatively low incidences in Western areas of Wales such as Ceredigion, Pembrokeshire, and Carmarthenshire. This variability could be related to a number of factors, including engagement with medical services in rural communities, a reflection of the low numbers of cases or the limitations of incomplete capture or misclassification of diagnoses and procedures that can occur. Nonetheless, geographic variability of microtia incidence has been previously shown in studies of the USA, Germany, Australia, England, and France (18).

Although our study population has a homogeneous ethnic population [94.1% Caucasian (47)], there is a wide spectrum of socioeconomic deprivation, offering useful insight for epidemiological analyses. In this study, the incidence of microtia appeared to be largely uniform in distribution between WIMD quintiles, with no statistically significant association observed between incidence and deprivation. This contrasts with previously published associations between microtia incidence and maternal educational status (33, 39) and socioeconomic deprivation in Hispanic populations (40). To date, there are a very limited number of studies in which the association between microtia incidence and socioeconomic deprivation has been explored in European populations, and there are none to date from the UK. An Italian cohort study determined no significant association between maternal education (as a marker of deprivation) and microtia incidence (41). In the UK, studies have demonstrated no association between socioeconomic deprivation and craniofacial syndromes (42) but an association between deprivation and congenital deafness (42) and in Wales specifically, the incidence of orofacial clefts (43).

This study indicates that the observed regional variability in microtia incidence in Wales is independent of the influence of socioeconomic deprivation and given the homogeneity of the population is unlikely to be related to ethnicity.

An additional aim of this study was to calculate the proportion of patients who opt for autologous vs. prosthetic reconstruction and to determine whether any demographic differences or inequalities exist in these cohorts. Of the patients who had not been operated on to date, just over 65% were still too young to be surgical candidates, which has limitations for drawing definitive conclusions relating to reconstructive modality.

Of the 48 patients who had surgery, the majority (73%) opted for autologous reconstruction. The median age of autologous ear reconstruction surgery was nine in our study, in line with UK guidelines (9, 23, 25), and the accepted wisdom that the ear is ~90% of its adult size by 8–10 years of age. This correlates with the age range in which children develop the self-awareness and psychosocial experiences to contribute to surgical decision making and when the rib cage is developed enough to allow harvest and carving with relative ease. The median age of alloplastic ear reconstruction was 7, which reflects both the younger age that these procedures can be performed, and the time needed to make a definitive reconstructive decision.

The patients opting for prosthetic reconstruction were from more deprived populations than the autologous group, with 41.7% of patients in the prosthetic cohort being in the most deprived WIMD quintile. Although not statistically significant, this is a clinically significant finding which warrants reflection. Autologous reconstruction is a complex surgical concept, and certainly in this study, is associated with a higher number of surgical procedures than alloplastic reconstruction. It is our duty as service providers to ensure that the information we provide to patients regarding surgical options is tailored, comprehensive and accessible to patients of all backgrounds and that inequalities in access to surgery are acknowledged, addressed and where possible, surmounted. Our previous work has shown that patient information sheets may not tailor to the average UK reader (44), with 1 in 6 individuals in the UK believed to have a literacy level below that expected of an 11 year old (46), and as a result of this, we are currently working on a bespoke patient information sheet for Microtia to accommodate the variation in literacy skills.

Our data demonstrates that the most deprived populations appeared to correlate with the lowest distances traveled for surgery. There are limitations in using the geodesic distance as a measure of travel, as it may markedly underestimate the distance patients endure as dictated by road and railway routes. It does, however, enable crude comparisons of the spectrum of distances traveled by patients from different geographical areas. Further work could be completed in the future to utilize network analyses utilizing road and travel data sources to evaluate more accurate distance and time point to point results, but this would have required additional permissions to access anonymized residential location identifiers for the cohort. The trend was toward the most deprived populations traveling the shortest distances, with the most deprived populations in WIMD quintiles 1 and 2 comprising 60.5% of those traveling <20 miles but only comprising 28.6% of patients traveling over 100 miles. Although there are no significant differences in the distance traveled for surgery between autologous and prosthetic groups, 60% of the patients who traveled to England were for autologous surgery with a median distance of >100 miles, despite this service being offered in Swansea. This reinforces the need for a robust and unified referral and treatment pathway for ear reconstruction in Wales, to address some of the inequalities in access to surgery and minimize the risk that ease of accessibility to one type of surgery may influence patient choice. This emphasizes the importance of a multidisciplinary team approach to enable information to be disseminated between clinicians treating microtia patients in Wales, which as per UK Care Standards should include a minimum of a reconstructive surgeon, otologist, audiologist, pediatrician with audiology interests, a clinical psychologist, specialist nurse, and anaplastologist (45).

## Conclusion

This study applied data-linkage methods to determine the 19-year incidence of microtia in a homogenous UK population (3.1 Million) to be greater than double previously reported values and highlighting that both temporal and geographical variation in microtia incidence exists. The majority of patients in this cohort opted for autologous reconstructive surgery, with the possibility that socioeconomic deprivation may bear an influence on the choice of reconstructive modality, which warrants further investigation, particularly where patients are required to travel long distances to access surgical intervention.

## Data Availability Statement

The original contributions presented in the study are included in the article/supplementary material, further inquiries can be directed to the corresponding author/s.

## Author Contributions

TJ, JG, TD, and IW contributed to the conception and design of the study. TJ, JG, RG, and AA were involved in the extraction and analysis of the data. TJ, JG, TD, MC, NW-J, RC, SK, PE, RL, and IW were involved in the interpretation of the data and drafting of the manuscript. All authors approve the final manuscript.

## Conflict of Interest

The authors declare that the research was conducted in the absence of any commercial or financial relationships that could be construed as a potential conflict of interest.
